# Targeted Next-Generation Sequencing in the Diagnosis of Facial Dysostoses

**DOI:** 10.3389/fgene.2020.580477

**Published:** 2020-11-11

**Authors:** Ewelina Bukowska-Olech, Anna Materna-Kiryluk, Joanna Walczak-Sztulpa, Delfina Popiel, Magdalena Badura-Stronka, Grzegorz Koczyk, Adam Dawidziuk, Aleksander Jamsheer

**Affiliations:** ^1^Department of Medical Genetics, Poznan University of Medical Sciences, Poznań, Poland; ^2^Postgraduate School of Molecular Medicine, Medical University of Warsaw, Warsaw, Poland; ^3^Centers for Medical Genetics GENESIS, Poznań, Poland; ^4^Department of Biometry and Bioinformatics, Institute of Plant Genetics Polish Academy of Sciences, Poznań, Poland

**Keywords:** facial dysostoses, mandibulofacial dysostoses, acrofacial dysostoses, pharyngeal arch, rare diseases, craniofacial development, targeted next-generation sequencing

## Abstract

**Background:**

Defects in the development of the first and second pharyngeal arches and their derivatives result in abnormal formation of the craniofacial complex, consequently giving rise to facial dysostoses (FDs). FDs represent a group of rare and highly heterogeneous disease entities that encompass mandibulofacial dysostoses (MFDs) with normal extremities and acrofacial dysostoses (AFDs) with limb anomalies in addition to craniofacial defects.

**Methods:**

We examined 11 families with variable clinical symptoms of FDs, in most of which only one member was affected. We applied two custom gene panels—first comprising 37 genes related to the genetic disorders of craniofacial development such as FDs (On-Demand AmpliSeq Thermo Fisher Scientific gene panel with two primer pools) and second composed of 61 genes and 11 single nucleotide variants (SNVs) known to be involved in the development of skull malformations, mainly in the form of craniosynostoses (SureSelect Agilent Technologies). Targeted next-generation sequencing (NGS) was performed using the Ion Torrent S5 platform. To confirm the presence of each detected variant, we have analyzed a genomic region of interest using Sanger sequencing.

**Results:**

In this paper, we summarized the results of custom targeted gene panel sequencing in the cohort of sixteen patients from 11 consecutive families affected by distinct forms of FDs. We have found three novel pathogenic variants in the *TCOF1* gene—c.2145_2148dupAAAG p.(Ser717Lys*fs*^∗^42), c.4370delA p.(Lys1457Arg*fs*^∗^118), c.83G>C p.(Arg28Pro) causing Treacher Collins syndrome type 1, two novel missense variants in the *EFTUD2* gene–c.491A>G p.(Asp164Gly) and c.779T>A p.(Ile260Asn) in two female patients affected by acrofacial dysostosis Guion-Almeida type, one previously reported–c.403C>T (p.Arg135Cys), as well as one novel missense variant–c.128C>T p.(Pro43Leu) in the *DHODH* gene in the male patient with Miller syndrome and finally one known pathogenic variant c.574G>T p.(Glu192^∗^) in the *SF3B4* gene in the patient with Nager syndrome.

**Conclusion:**

Our study confirms the efficiency and clinical utility of the targeted gene panel sequencing and shows that this strategy is suitable and efficient in the molecular screening of variable forms of FDs.

## Background

The human skull composes of neuro- and viscerocranium that both develop independently from one another. Neurocranium mainly forms from the first five somites and the unsegmented somitomeres rostral to the first somite, whereas, viscerocranium, namely facial skeleton, forms from pharyngeal arches (Jin et al., 2016).

Defects concerning embryonic development of the first and second pharyngeal arches and their derivatives result in facial dysostoses (FDs), which encompass a group of rare and extremely variable clinical disease entities. FDs have been subdivided into mandibulofacial dysostoses (MFDs) with normal extremities and acrofacial dysostoses (AFDs) with limb anomalies in addition to craniofacial malformations (Table 1; Trainor and Andrews, 2013; Wieczorek, 2013). The characteristic facial features of FDs involve hypoplasia of the maxilla, mandible, zygomatic arches, various anomalies of the external and middle ear, and other facial defects including coloboma of the lower eyelid with partial to total absence of eyelashes or dysmorphic features, such as down-slanting palpebral fissures. Limb defects represent a broad spectrum of post- or preaxial abnormalities, including polydactyly, oligodactyly, and brachydactyly (Ogilvy-Stuart and Parsons, 1991; McDonald and Gorski, 1993; Wieczorek et al., 2009; Ng et al., 2010; Rainger et al., 2012; Czeschik et al., 2013; Trainor and Andrews, 2013; Wieczorek, 2013). Additional clinical signs, which may be present comprise microcephaly, cleft palate, oligodontia, defects of the internal organs, short stature, psychomotor delay, and intellectual disability (Guion-Almeida et al., 2006; Wieczorek, 2013).

**TABLE 1:** Clinical classification of human facial dysostoses.

**Type of facial dysostosis**	**Subtype of facial dysostosis**	**OMIM**	**Gene/locus**	**Inheritance**
Mandibulofacial dysostosis	Bauru	604830	nd	nd
Mandibulofacial dysostosis	Burn-McKeown	608572	*TXNL4A*	AR
Mandibulofacial dysostosis	Hedera-Toriello-Petty	608257	nd	nd
Mandibulofacial dysostosis	Toriello	301950	nd	nd
Mandibulofacial dysostosis	Treacher Collins type 1	154500	*TCOF1*	AD
Mandibulofacial dysostosis	Treacher Collins type 2	613717	*POLR1D*	AD, AR
Mandibulofacial dysostosis	Treacher Collins type 3	248390	*POLR1C*	AD, AR
Mandibulofacial dysostosis	Verloes-Lesenfants	302562	nd	nd
Mandibulofacial dysostosis	Zhang	–	Duplication 1p36.33	nd
			Duplication 1q21.2-q22	
Acrofacial dysostosis	Arens	–	nd	nd
Acrofacial dysostosis	Bates	–	nd	nd
Acrofacial dysostosis	Catania	101805	nd	AD
Acrofacial dysostosis	Cincinnati	616462	*POLR1A*	AD
Acrofacial dysostosis	Guion-Almeida	610536	*EFTUD2*	AD
Acrofacial dysostosis	Karaman-Kavecci	–	nd	nd
Acrofacial dysostosis	Kennedy-Teebi	–	nd	AR
Acrofacial dysostosis	Kelly	–	nd	AR/XR
Acrofacial dysostosis	Macena Sobreira	–	nd	nd
Acrofacial dysostosis	Miller syndrome	263750	*DHODH*	AR
Acrofacial dysostosis	Nager syndrome	154400	*SF3B4*	AD
Acrofacial dysostosis	Patterson-Stevenson and Fontaine	183700	nd	nd
Acrofacial dysostosis	Palagonia	601829	nd	AD
Acrofacial dysostosis	Reynolds	–	*Microdeletion 16p13.3?*	nd
Acrofacial dysostosis	Richieri-Costa-Pereira	268305	*EIF4A3*	AR
Acrofacial dysostosis	Rodriguez	201170	nd	AR
Acrofacial dysostosis	Weyers	193530	*EVC, EVC2*	AD

*AD, autosomal dominant; AR, autosomal recessive; nd, no data.*

Importantly, FDs present a high clinical heterogeneity, including a marked inter- and intra-familial variability of their phenotypic expression what in turn frequently impedes reaching an exact clinical diagnosis and its confirmation at a molecular level (Dixon et al., 2004; Vincent et al., 2016). Consequently, many patients affected by FDs remain molecularly undiagnosed and bereft of reliable genetic counseling, prenatal and preimplantation testing, but also of a proper and unequivocal clinical classification (Ng et al., 2010; Bernier et al., 2012; Lines et al., 2012; Fan et al., 2019).

## Materials and Methods

All procedures were performed under the ethical standards of the institutional and national research committee and with the 1964 Helsinki declaration and its later amendments or comparable ethical standards. We granted ethics approval from the Institutional Review Board of Poznan University of Medical Sciences (no. 741/17 and 742/17 obtained on 22^nd^ June 2017). We examined 11 families with variable clinical symptoms of FDs, in most of which only one member was affected. We extracted genomic DNA from the peripheral blood lymphocytes using the MagCore HF16 Automated Nucleic Acid Extractor and quantified each gDNA using the Agilent Technologies TapeStation 2200 and Qubit.

### Targeted Next-Generation Sequencing

### Facial Dysostoses Panel

We designed the custom On-Demand AmpliSeq (Thermo Fisher Scientific) gene panel with two primer pools and performed targeted sequencing of 37 genes related to the genetic disorders of craniofacial development, including FDs (patients 1.4, 1.6, 2–4, 6 and 7; Supplementary Table 1). The gene panel was 145.51 kb in size and comprised 761 amplicons, intervals sequenced for each gene were shown in Supplementary Table 2. The barcoded genomic DNA libraries were constructed according to the ‘manufacturer’s sample preparation protocol (Ion AmpliSeq Library Kit 2.0; On-Demand Panels). Pooled libraries were subjected to emulsion PCR on Ion Chef^TM^ instrument using the Ion 520^TM^ and 530^TM^Kit, according to the standard protocol and sequenced on the Ion Torrent S5 platform.

### Craniosynostosis Panel

The custom SureSelect (Agilent Technologies) gene panel comprised of 61 genes and 11 SNVs known to be involved in the development of craniofacial malformations, particularly craniosynostosis (CS), was designed (Supplementary Table 3). Intervals sequenced for each gene were shown in Supplementary Table 4. Targeted sequencing, encompassing 225.709 kb was performed for patient 5. We prepared the NGS library using hybridized capture-based target enrichment approach and sequenced it on the Ion Torrent S5 platform (Bukowska-Olech et al., 2020).

### Next-Generation Sequencing Data Analysis

The present analysis follows the approach previously described in Bukowska-Olech et al. (2020). Briefly, the reads were cleaned and aligned to GrCh37 human reference sequence using the TorrentBrowser 5.0.4 software (Thermo Fisher Scientific). The alignments were further processed using IonReporter 5.2 pipeline (Thermo Fisher Scientific), which incorporated variant calling with Torrent Variant Caller 5.4–11. Each variant was examined based on four criteria (read depth− ≥ 20, strandedness− ≤ 4:1, PHRED score ≥ 30 and variant frequency ≥ 0.15). The coverage of individual genes and exons was performed using bedtools 2.27.1 (*coverage* subcommand) against panel-specific BED files defining coding parts of canonical transcripts for each constituent gene (RefSeq mapped on UCSC hg19 reference; 5 bp padding around each exon included; Supplementary Tables 5–8).

Previous clinical annotation of called variants was obtained from Human Gene Mutation Database (HGMD) Professional (accessed on 14/02/2019), ClinVar (accessed on 27/07/2019) and dbSNP (accessed on 27/07/2019). The predictions for SIFT, PolyPhen, and PhyloP (46-way) tools were retrieved from IonReporter result files. Population allele frequencies were annotated by Ensembl/Variant Effect Predictor (software version 94, database version 94); additionally, gnomAD database was queried for homozygosity/heterozygosity of individual variants (version 2.1.1 of both exome and genome subsets; accessed on 27/07/2019 using tabix 1.5 software). The scores for Combined Annotation Dependent Depletion were obtained from CADD webserver (v1.4, accessed on 27/07/2019). MutationTaster results were obtained using the “query chromosomal position” option of the public webserver (accessed on 27/07/2019).

### Sanger Sequencing

To confirm the presence of each pathogenic or likely pathogenic variant, as well as of variant of uncertain significance (VUS), we have analyzed the genomic region of interest using Sanger sequencing. The online available Primer3 tool was used to design specific primers for the amplification. The PCR reactions and PCR products purification were carried out following standard protocols. Sanger sequencing was performed using dye-terminator chemistry (kit v.3, ABI 3130XL) and run on automated sequencer Applied Biosystems Prism 3700 DNA Analyzer. Variants were annotated against the reference sequences: *DHODH*—NG_016271.1, NC_000016.10, NM_001361.4, NP_001352.2; *EFTUD2*—NG_032674.1, NC_000017.11, NM_004247.3, NP_004238.3; *SF3B4*—NG_032777.1, NC_000001.11 NM_005850.4, NP_005841.1, *TCOF1*—NG_011341.1, NC_000005.10, NM_001135243.1, NP_001128715.1 following the Human Genome Variation Society (HGVS) nomenclature guidelines (den Dunnen et al., 2016; Table 2).

**TABLE 2:** Summary of pathogenic variants reported in this paper.

	**Gene**	**HGVS genomic**	**HGVS chromosomal**	**HGVS coding**	**HGVS protein level**	**Location**	**ACMG Classification**	**CADD**	**SIFT**	**PolyPhen**	**MutationTaster**
Family 1	*TCOF1*	NG_011341.1: g.18787_18790dup	NC_000005.10: g.150376425_ 150376428dup	NM_001135243.1: c.2145_2148dup	NP_001128715.1: p.(Ser717Lysfs*42)	Exon 10	Pathogenic	15.57	n/a	n/a	Disease causing
Patient 2	*TCOF1*	NG_011341.1: g.191G>C	NC_000005.10: g.150357829G>C	NM_001135243.1: c.83G>C	NP_001128715.1: p.(Arg28Pro)	Exon 1	Variant of Uncertain Significance	33	n/a (0^∧^)	0.998	Polymorphism
Patient 3	*TCOF1*	NG_011341.1: g.40740_40740delA	NC_000005.10: g.150398381delA	NM_001135243.1: c.4370delA	NP_001128715.1: p.(Lys1457Argfs*118)	Exon 25	Pathogenic	31	n/a	n/a	Disease causing
Patient 4	*SF3B4*	NG_032777.1: g.1837G>T	NC_000001.11: g.149926508C>A	NM_005850.4: c.574G>T**^#^**	NP_005841.1: p.(Glu192*)**^#^**	Exon 3	Pathogenic	38	n/a	n/a	Disease causing
Patient 5	*EFTUD2*	NG_032674.1: g.16569A>G	NC_000017.11: g.44883094T>C	NM_004247.3: c.491A>G	NP_004238.3: p.(Asp164Gly)	Exon 6	Likely Pathogenic	25	0.23	0.095	Disease causing
Patient 6	*EFTUD2*	NG_032674.1: g.23639T>A	NC_000017.11: g.44876024A>T	NM_004247.3: c.779T>A	NP_004238.3: p.(Ile260Asn)	Exon 10	Likely Pathogenic	27.2	0.0	0.972	Disease causing
Patient 7	*DHODH*	NG_016271.1: g.3569C>T	NC_000016.10: g.72012156C>T	NM_001361.4: c.128C>T	NP_001352.2: p.(Pro43Leu)	Exon 2	Variant of Uncertain Significance	26.1	1.0	0.0	Disease causing
	*DHODH*	NG_016271.1: g.6054C>T	NC_000016.10: g.72014641C>T	NM_001361.4: c.403C>T**^#^**	NP_001352.2: (p.Arg135Cys)**^#^**	Exon 3	Pathogenic	32	1.0	0.0	Disease causing

*American College of Medical Genetics (ACMG) classification was obtained through VarSome online available tool (accessed on 23/08/2020). PolyPhen and SIFT scores were obtained through IonReporter pipeline and cross-referenced with Variant Effect Predictor (VEP) results. **^#^** variant reported previously, ∧ score reported through Ensembl VEP.*

## Results

### Clinical Assessment

We recruited 11 consecutive families in whom 16 individuals were affected by various forms of FDs. Four families (no. 1–3, 6) received the clinical diagnosis of MFDs defined as hypoplastic maxilla, mandible, and zygomatic arches with down-slanting palpebral fissures. Six families (no. 4, 8–11) were clinically diagnosed with AFDs since the affected individuals showed not only craniofacial but also limb involvement. In one family (family 5), we did not recognize FD based on the dysmorphological assessment. The detailed clinical data of all screened patients is set forth in Supplementary Data Sheet 1.

### Genetic Analyses

Targeted NGS allowed us to reach an exact diagnosis of FDs in seven out of eleven families (no. 1–7). Among them, we identified six novel and two previously reported variants in the following genes–*TCOF1*, *SF3B4*, *EFTUD2*, and *DHODH* (Table 2). The mean coverage for FDs panel ranged from 214 to 430 reads per gene, whereas for CS panel from 272 to 488 reads per gene (Supplementary Tables 5, 7).

**FIGURE 1:**
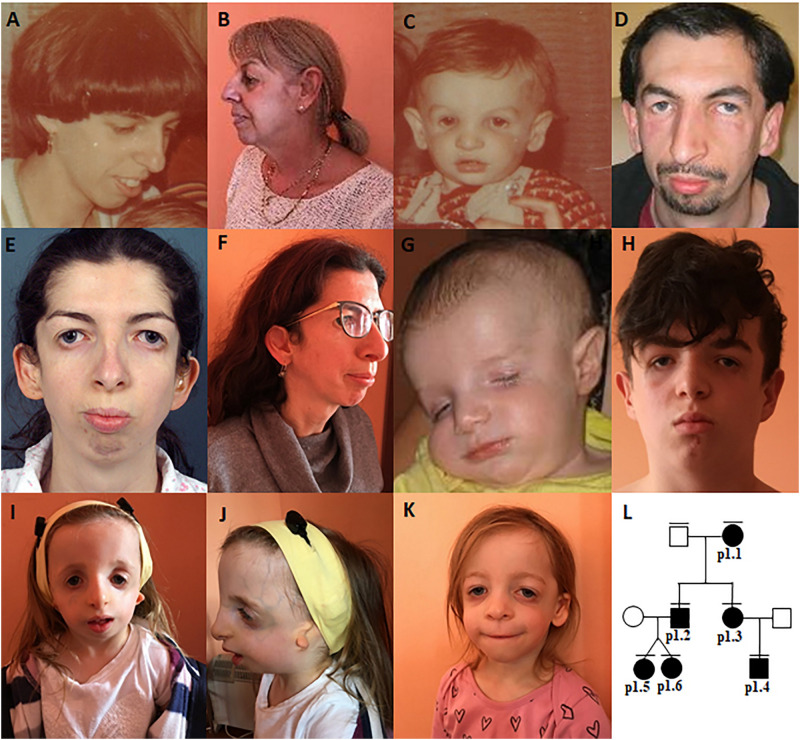
Clinical characteristics of family 1–patient 1.1 **(A,B)**, patient 1.2 **(C,D)**, patient 1.3 **(E,F)**, patient 1.4 **(G,H)**, patient 1.5 **(I,J)**, patient 1.6 **(K)**, The pedigree **(L)**.

**FIGURE 2:**
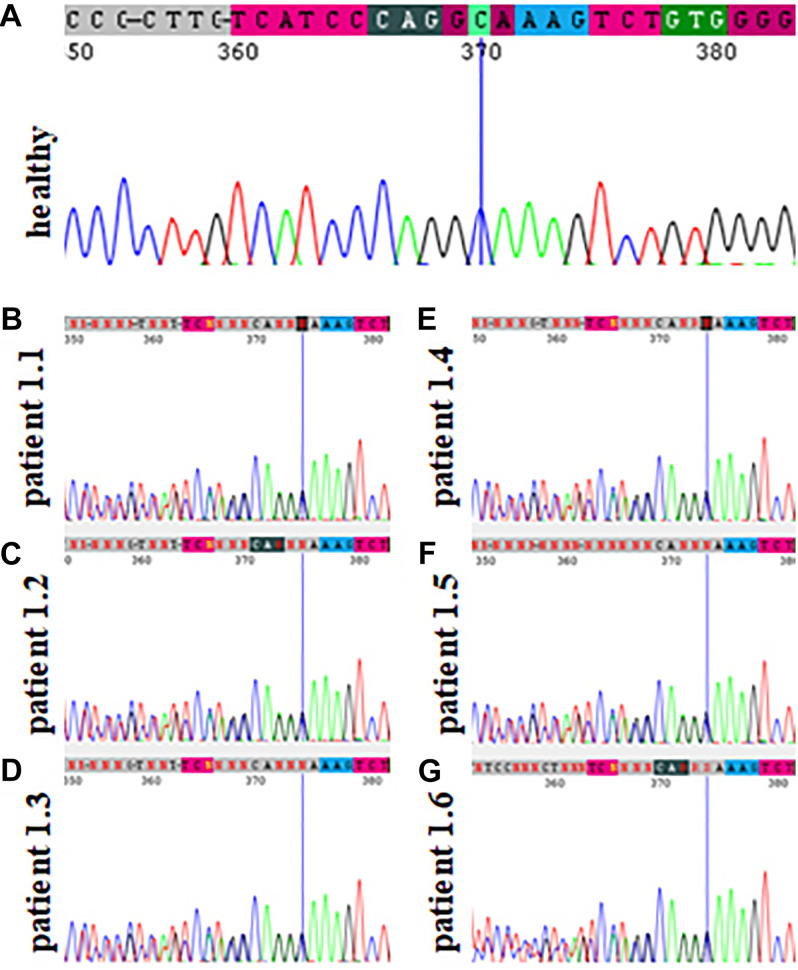
Sanger sequencing results of family 1 showing the absence of the pathogenic variant c.2145_2148dupAAAG p.(Ser717Lysfs*42) within the *TCOF1* gene in a healthy family member **(A)** and its presence in patients affected by Treacher Collins syndrome **(B–G)**.

**FIGURE 3:**
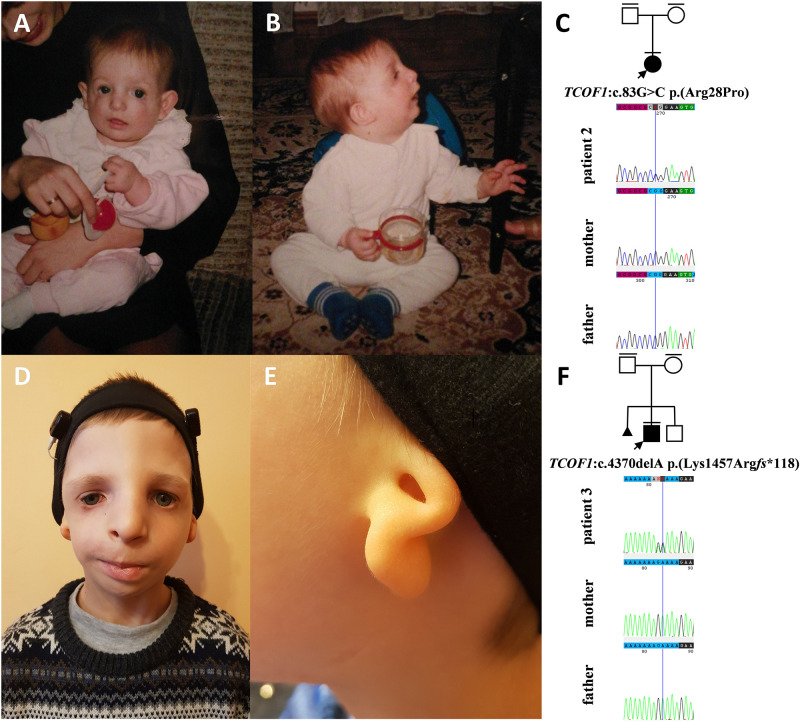
Clinical characteristics and segregation results of patient 2 **(A–C)** and 3 **(D–F)** affected by Treacher Collins syndrome. Patient 2 carries a heterozygous variant c.83G>C p.(Arg28Pro) within the *TCOF1* gene, which occurred *de novo* **(C)** whereas patient 3 carries a heterozygous variant c.4370delA p.(Lys1457Argfs*118) within the *TCOF1* gene, which also occurred *de novo*.

**FIGURE 4:**
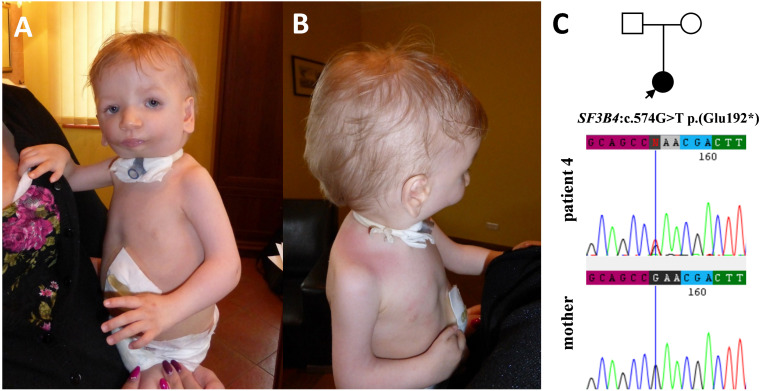
Clinical characteristics of patient 4 affected by Nager syndrome **(A,B)**. Patient 4 carries a heterozygous variant c.574G>T p.(Glu192*) within the *SF3B4* gene, which was excluded in her healthy mother **(C)**.

**FIGURE 5:**
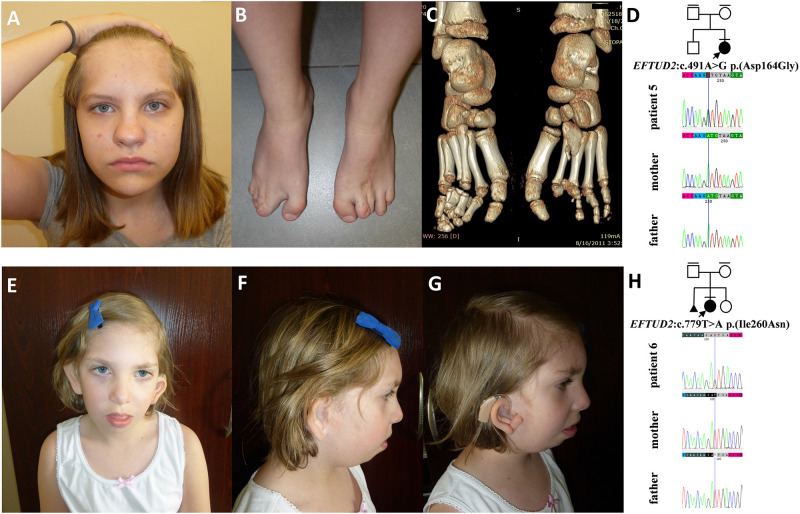
Clinical characteristics and segregation results of patient 5 **(A–D)** and 6 **(E–H)** affected by acrofacial dysostosis Guion-Almeida type. Patient 5 carries a heterozygous variant c.491A>G p.(Asp164Gly) within the *EFTUD2* gene, which occurred *de novo* **(D)** whereas patient 6 carries a heterozygous variant c.779T>A p.(Ile260Asn) within the *EFTUD2* gene, which also occurred *de novo* **(H)**.

**FIGURE 6:**
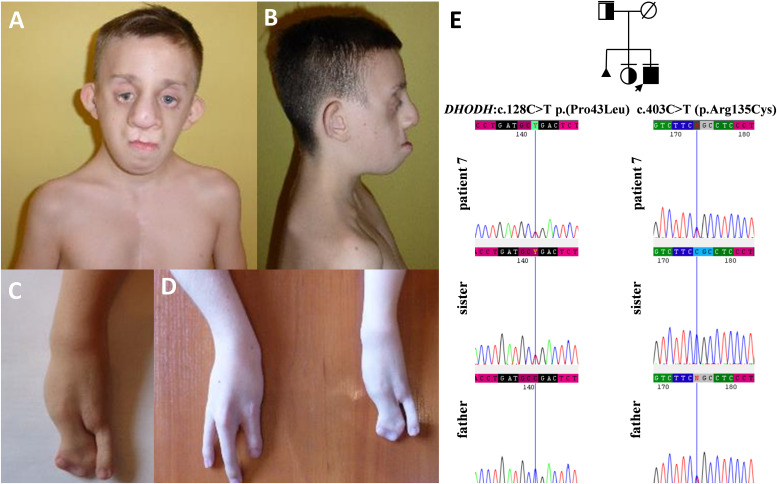
Clinical characteristics **(A–D)** and segregation results **(E)** of patient 7 affected by Miller syndrome. Patient 7 carries c.403C>T (p.Arg135Cys) and c.128C>T p.(Pro43Leu) missense variants, both located in trans orientation in the *DHODH* gene **(E)**.

In families no 1–3, we found three novel heterozygous variants in the *TCOF1* that gave rise to the most well-characterized MFD, i.e., Treacher Collins syndrome (TCS) type 1 (TCS1, MIM:154500). The major clinical features of all affected individuals were summarized in Table 3. The rest of the diagnosed patients was affected by different types of AFDs. In a sporadic female case from family no 4, we identified the heterozygous variant in the *SF3B4* gene causing Nager syndrome (MIM:54400). Two other sporadic female cases from families no 5 and 6 harbored *de novo* heterozygous variants within the *EFTUD2* gene and presented with variable symptoms of acrofacial dysostosis Guion-Almeida type (AFDGA, MIM:610536). The comparison of their clinical features was shown in Table 4. Finally, in a male proband from family no 7, we diagnosed Miller syndrome (MIM:263750), as we found two biallelic variants in the *DHODH* gene. Importantly, none of the detected alterations was present in gnomAD or ExAC databases or found in 200 ethnically matched control alleles from our in-house, population-specific database. Segregation analysis of the causative variants performed in families 1–6 was consistent with the expected autosomal dominant inheritance pattern (Figures 2, 3C,F, 4C, 5D,H), whereas in family 7 with autosomal recessive pattern (Figure 6E). The results of molecular screening and segregation analyses were shown in Figures 1–6 and Supplementary Figures 1A–I.

**TABLE 3:** Summary of clinical data in reported patients with Treacher Collins syndrome (TCS).

	**Patient 1.1 (Figures 1 A,B)**	**Patient 1.2 (Figures 1C,D)**	**Patient 1.3 (Figures 1E,F)**	**Patient 1.4 (Figures 1G,H)**	**Patient 1.5 (Figures 1I,J)**	**Patient 1.6 (Figure 1K)**	**Patient 2 (Figures 3A,B)**	**Patient 3 (Figures 3D,E)**
Gender	F	M	F	M	F	F	F	M
Mandibular hypoplasia	+	+	+	+	+	+	+	+
Maxillary hypoplasia	+	+	+	+	+	+	+	+
Downward slanting palpebral fissures	+	+	+	+	+	+	+	+
Partial absence of eyelids	–	−	−	−	+	+	−	−
Microtia	−	−	−	−	+	+	−	+
Conductive hearing loss	+	+	+	+	+	+	+	+
Cleft lip/palate	−	−	−	−	+	−	−	+

**TABLE 4:** Summary of clinical data in reported patients with acrofacial dysostoses Guion-Almeida type (AFDGA).

	**Patient 5**	**Patient 6**
Gender	F	F
Intellectual impairment	−	+
Delayed psychomotor development	−	+
Delayed speech development	−	+
Epilepsy	−	+
Microcephaly	−	+
Trigonocephaly	+	+
Midface hypoplasia	−	+
Malar hypoplasia	−	+
Micrognathia	−	+
Buccal tags	−	+
Microtia	−	−
Preauricular tag	−	+
Preauricular pit	−	+
Low-set ears	−	+
Dysplastic ears	−	+
Conductive hearing loss	−	+
Upslanting palpebral fissures	−	−
Downslanting palpebral fissures	−	+
Epicanthal folds	−	−
Choanal atresia	−	−
Upturned nose	+	−
Short nose	−	+
Cleft palate	−	−
Atrial septal defect	−	+
Breathing difficulties due to choanal atresia	−	−
Esophageal atresia	−	−
Preaxial polydactyly	+	−
Proximally placed thumbs	−	−

## Discussion

Before the advent of NGS, identification of the underlying causative genetic lesions in individuals affected by molecularly complex genetic syndromes such as FDs was often difficult or sometimes impossible (Fan et al., 2019). In an excellent review on human facial dysostoses, Wieczorek emphasized that the current classification of FDs bases mostly on the clinical characteristics of the facial features as well as the presence or absence of additional symptoms (Table 1; Wieczorek, 2013). The molecular confirmation of FDs is unquestionably crucial not only for reliable genetic counseling, prenatal and preimplantation testing, but also for a proper and unequivocal clinical classification.

In this paper, we summarized the results of the custom targeted gene panel sequencing in a group of 16 patients from 11 consecutive families affected by distinct forms of FDs that were consulted in our genetic clinic. In this cohort, we detected three novel heterozygous variants within the *TCOF1* gene, two of which were frameshifts: c.2145_2148dupAAAG p.(Ser717Lys*fs*^∗^42) (family 1; Figure 2), c.4370delA p.(Lys1457Arg*fs*^∗^118) (family 3; Figure 3F), while one was a missense alteration c.83G>C p.(Arg28Pro) (family 2; Figure 3C). *In silico* analyses predicted that frameshifts truncating Treacle protein at codons 717 and 1457 were pathogenic, whereas a missense variant p.(Arg28Pro) was initially classified as a VUS (Table 2). The results of segregation analysis performed in family 2 showed that the variant occurred *de novo*, suggesting its contribution to the patient’s phenotype (Figure 3C). The rest of the diagnosed individuals was affected by various types of AFDs. Among them, we identified one previously reported nonsense heterozygous pathogenic variant in the *SF3B4* gene–c.574G>T p.(Glu192^∗^) (family 4; Figure 4C), two novel missense likely pathogenic heterozygous variants in the *EFTUD2* gene, which occurred *de novo*–c.491A>G p.(Asp164Gly) (family 5; Figure 5D), c.779T>A p.(Ile260Asn) (family 6; Figure 5H), and one previously reported–c.403C>T (p.Arg135Cys) (evaluated as pathogenic), as well as one novel—c.128C>T p.(Pro43Leu) missense variants (evaluated as VUS), both located in trans orientation in the *DHODH* gene (family 7; Figure 6E).

Pathogenic variants in the *TCOF1* but also the *POLR1D* and *POLR1C* genes have been previously associated with Treacher Collins syndrome (TCS) types 1–3, respectively (Dixon et al., 1996; Dauwerse et al., 2011; Schaefer et al., 2014). According to the current review of medical literature, in the majority of patients (i.e., 77–89%) causative alterations are found within the *TCOF1* gene (encoding Treacle protein). Consistently with these results, we detected all three causative variants in the *TCOF1* (families no 1–3), and not in other TCS-related genes (Dauwerse et al., 2011). TCS is characterized by malar hypoplasia, micrognathia, down-slanting palpebral fissures, atresia of the external auditory canals, anomalies of the middle ear ossicles, and bilateral conductive hearing loss (Stovin et al., 1960; Phelps et al., 1981; Trainor et al., 2009; Vincent et al., 2016). Nevertheless, the phenotype often varies not only between, but also within the affected families. We have confirmed that in cases with TCS1 there is no clear genotype-phenotype correlation and that the expressivity of the clinical symptoms shows considerable intra- and interfamilial variability suggesting the influence of additional contributors (Table 3). However, the presence of modifying factors, probably involved in the process of mRNA level regulation, requires further research.

In the vast majority of families affected by AFDs, we have suspected Nager syndrome, which surprisingly was proved genetically only in one individual (patient 4). The families 5 and 7 that were misdiagnosed with Nager syndrome turned out to represent AFDGA (family 6) and Miller syndrome (family 7). Nager syndrome, along with AFDGA and Miller syndrome, share similar facial and skeletal features, hence the unambiguous diagnosis based only on the clinical symptoms may be uncertain (Trainor and Andrews, 2013; Wieczorek, 2013). Variants in the *SF3B4* gene that encodes a splicing factor 3B subunit 4 are found in about half of the patients clinically diagnosed with Nager syndrome (Bernier et al., 2012; Czeschik et al., 2013). The main phenotypic features of this condition involve facial characteristics similar to TCS and preaxial limb defects such as hypoplasia or aplasia of the radius and thumbs, radioulnar synostosis or triphalangeal thumbs (Bowen and Harley, 1974; Meyerson et al., 1977; Opitz, 1987; McDonald and Gorski, 1993; Wieczorek, 2013). A nonsense alteration p.(Glu192^∗^) within splicing factor 3B subunit 4, which occurred in patient 4, was firstly described by Czeschik et al. in a female patient with Nager syndrome, who manifested very similar symptoms to patient 4. Both individuals presented with extreme hypoplasia of the mandible, micrognathia, hypoplastic thumbs, bilateral limited elbow extension, hearing loss and delayed psychomotor development, with absent speech due to severe hypoplasia of the mandibulofacial structures (Czeschik et al., 2013). Additionally, patient 4 had an ectopic left kidney, which was located in the pelvis.

In families no. 5 and 6, we have found novel heterozygous variants in *EFTUD2*, the gene encoding elongation factor Tu GTP binding domain containing 2, resulting in AFDGA (Guion-Almeida et al., 2006; Lines et al., 2012). This entity was initially classified as mandibulofacial dysostosis Guion-Almeida type (MFDGA). Nevertheless, due to the occurrence of limb anomalies in some patients, it was reclassified to AFDs (Voigt et al., 2013). The clinical spectrum of AFDGA is broad and consists of microcephaly, postnatal-onset growth deficiency, choanal atresia, heart and gastrointestinal anomalies, preaxial polydactyly, slender fingers, and proximally placed thumbs (Guion-Almeida et al., 2006; Wieczorek et al., 2007, 2009; Lines et al., 2012; Gordon et al., 2015). Very recently, new craniofacial phenotypes, i.e., metopic craniosynostosis (CS) and cleft lip and palate, were reported in two individuals carrying heterozygous variants–c.2466+1G>A in intron 24 and c.2333C>A in exon 23 of *EFTUD2*. These cases were initially suspected of having an alternative craniofacial syndrome and were eventually diagnosed with MFDGA (Lacour et al., 2019). Similarly, patient 5 also presented with very unusual craniofacial features, including metopic synostosis and high forehead that were not suggestive for AFDGA.

Finally, in patient 7, we described compound heterozygous variants in *DHODH* gene. Biallelic variants in *DHODH* gene result in defects in pyrimidine biogenesis and give rise to Miller syndrome that has an autosomal recessive inheritance. Limb anomalies include postaxial abnormalities, such as the absence of fifth digits with or without shortening and deviation of forearms with ulnar and radial hypoplasia, and syndactyly (Genee, 1969; Ogilvy-Stuart and Parsons, 1991; Chrzanowska and Fryns, 1993; Ng et al., 2010). The first pathogenic missense variant (p.Arg135Cys) has been reported previously in six individuals affected by Miller syndrome who were compound heterozygous for (p.Arg135Cys) in combination with one of the following alterations–c.155A>G (p.Glu52Gly), c.1036C>T (p.Arg346Trp), and c.1175A>G (p.Asp392Gly). All those patients, including patient 7 (Figures 6A–D), presented with strikingly similar features, encompassing severe micrognathia, cleft lip and/or palate, as well as hypoplasia or aplasia of the postaxial rays of the limbs (Ng et al., 2010; Al Kaissi et al., 2011; Rainger et al., 2012). The second missense variant c.128C>T p.(Pro43Leu) identified by us was novel.

The diagnostic success rate of the presented approach achieved 64%. Undiagnosed cases (families 8–11) may harbor other types of causative alterations that cannot be detected by targeted NGS, such as deep intronic or regulatory mutations affecting genes included in custom gene panels. Alternatively, causative mutations may be located elsewhere in the genome and not in the genes known to be involved in the pathogenesis of FDs. Therefore, in cases without the exact genetic diagnosis, another type of molecular investigation, including whole-genome sequencing (WGS) should be considered. Indeed, the introduction of WGS in the molecular diagnostics of FDs requires advanced *in silico* and functional analyses, which are both necessary for the interpretation and proving of pathogenicity of genomic alterations (Smedley et al., 2016; Bodea et al., 2018; Posey, 2019). The proportion of molecularly undiagnosed patients, however, clearly suggests that some of the genetic lesions, causative for FDs, lie within non-coding DNA, and could be picked up only by WGS (Kindberg and Bush, 2019).

In conclusion, with this report, we demonstrated the clinical utility of NGS targeted gene panel approach as a valuable and cost-effective first-tier testing in the molecular analysis of patients presenting with FDs. We described the clinical phenotypes of seven families showing distinct forms of FDs and identified six novel and two previously described pathogenic variants. Consequently, we highlighted the need for molecular confirmation of FDs, which is crucial for a reliable genetic counseling, an early prenatal and preimplantation testing, but also a proper and unequivocal clinical classification.

## Data Availability Statement

Data generated during this study are included in this published article and its supplementary material. BAMs files were submitted on Sequence Read Archive (SRA) NCBI (SRA submission: PRJNA663125, https://www.ncbi.nlm.nih.gov/bioproject/PRJNA663125.

## Ethics Statement

The studies involving human participants were reviewed and approved by the Bioethics Committee at Poznan University of Medical Sciences (no. 741/17 and 742/17) according to the Good Clinical Practice and Polish law. All patients and their parents agreed to participate in this study. Written informed consent to participate in this study was provided by the participants’ legal guardian/next of kin. Written informed consent was obtained from the minor(s)’ legal guardian/next of kin for the publication of any potentially identifiable images or data included in this article.

## Author Contributions

AJ, AM-K, and MB-S recruited and clinically diagnosed the patients. EB-O designed the study, performed, analyzed the molecular data, and wrote the manuscript. JW-S participated in data analysis. DP participated in a part of experimental procedures. GK participated in a part of data analysis. AD participated in a part of experimental procedures. AJ designed the study and critically revised and corrected the manuscript. All authors read and approved the final manuscript.

## Conflict of Interest

The authors declare that the research was conducted in the absence of any commercial or financial relationships that could be construed as a potential conflict of interest.
