# Supplementary figures and images for: Targeted Next-Generation Sequencing in the Diagnosis of Facial Dysostoses
Source: Front Genet. 2020 Nov 11;11:580477. doi: 10.3389/fgene.2020.580477 (PMC7686794; doi:10.3389/fgene.2020.580477)

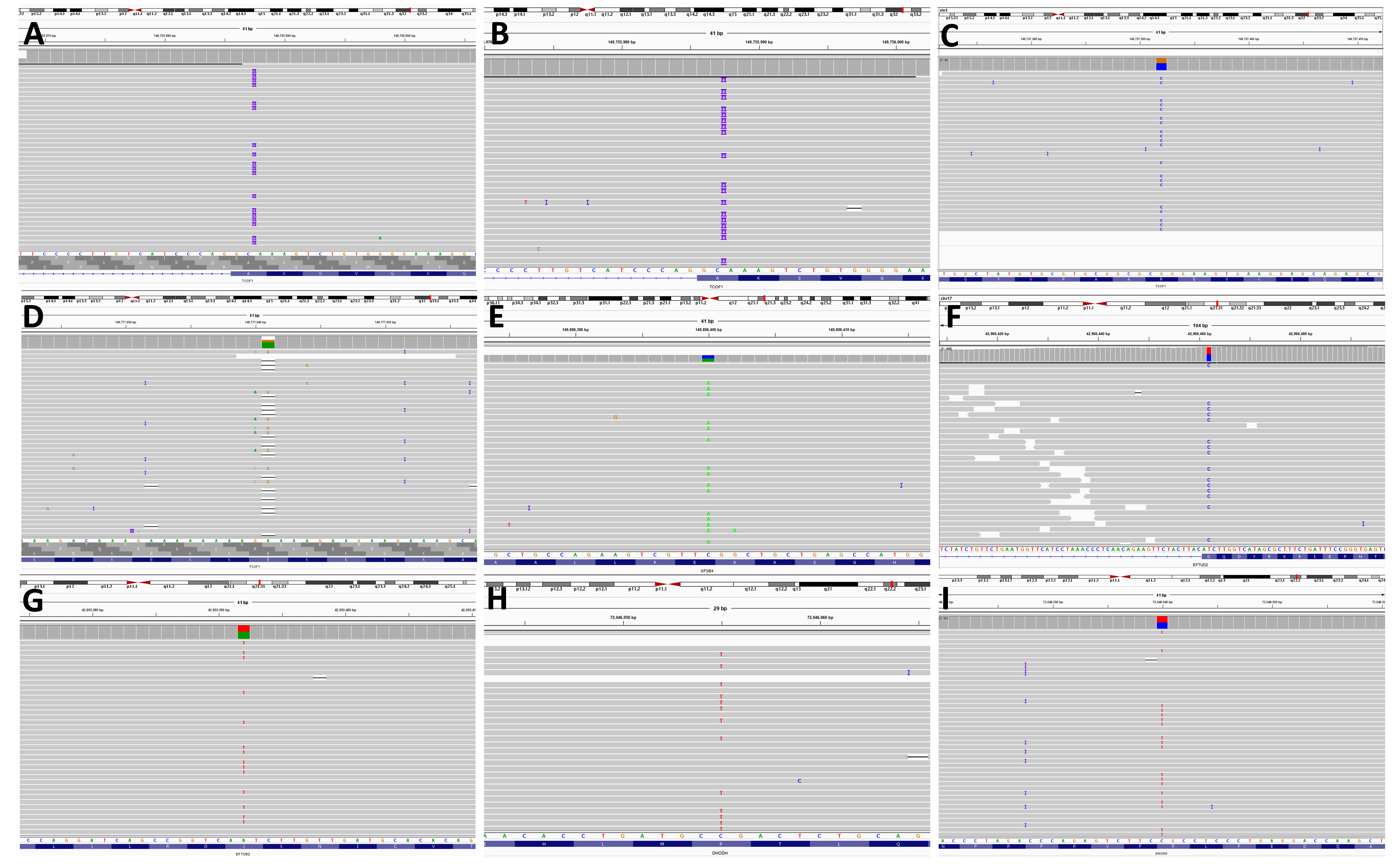

Supplement: Supplementary file 1 [file Image_1.tif]
