# Supplementary material for: Targeted Next-Generation Sequencing in the Diagnosis of Facial Dysostoses
Source: Front Genet. 2020 Nov 11;11:580477. doi: 10.3389/fgene.2020.580477 (PMC7686794; doi:10.3389/fgene.2020.580477)
Supplement: Supplementary file 10 [file Data_Sheet_1.docx]

**Clinical data
Family 1 (six affected members)**

Family 1 was a three-generation pedigree in which six members (patients 1.1, 1.2, 1.3, 1.4, 1.5, 1.6) were clinically diagnosed with TCS with a variable phenotypic expression (Figure 1, Table 3). To find the molecular cause of the syndrome, we analyzed two most distant relatives (patient 1.4 and 1.6) using targeted NGS.

Patient 1.4, the index patient from family 1, was a boy born in the 40^th^ week of gestation from 1^st^ uncomplicated pregnancy to unrelated parents – to the mother affected by TCS (patient 1.3) and the father affected by profound hearing loss. His body mass was 3900 g (75^th^-90^th^ percentile), length 56 cm (above 97^th^ percentile), Apgar score was 9-10 at 1,3’. Genetic consultation revealed typical appearance suggestive for TCS – micrognathia, retrognathia, down-slanting palpebral fissures, asymmetry of the orbits and bilateral hearing loss (50-60 dB). At the age of 13 years, his weight was 72 kg (90-97^th^ percentile) and height 1.70 m (90^th^ percentile). Patient 1.5&1.6 were 3.5 years old female twins from 1^st^ dichorionic and diamniotic pregnancy complicated by maternal arterial hypertension. They were born by Caesarian section in the 34-35^th^ week of gestation to a healthy mother and a father affected by TCS (patient 1.2). The birth parameters of patient 1.6 were as follows: weight 2230 g (25-50^th^ percentile), length 48 cm (75-90^th^ percentile), head circumference 32 cm (50^th^ percentile), Apgar score was 9-9 at 1,3’. The girl presented dysmorphic features – dysplastic right ear, hypoplastic mandible, subtle retrognathia, and triangular face, discrete down-slanting of the palpebral fissures, low-short eyelids and partial absence of lower eyelashes. The transfontanellar and abdominal ultrasound scans were unremarkable. Unilateral conductive hearing loss was noted (60 dB). At the 3.5 years, her psychomotor and intellectual development were normal.

After discovering a novel pathogenic variant within the *TCOF1* gene c.2145_2148dupAAAG p.(Ser717Lys*fs**42), we performed Sanger sequencing in four other affected relatives (Figure 2, Additional File 3). The results confirmed the clinical diagnosis of TCS1.

**Family 2 (sporadic case)**

Patient 2 – a 25 years old female was born to healthy and unrelated parents. Birth weight was 3300 g (50-75^th^ percentile), length 54 cm (above 97^th^ percentile), HC 35 cm (75^th^-90^th^ percentile), Apgar score was 10 at 1’. At the age of 4 months, she was clinically diagnosed with TCS, whereas at the age of 12 months, hearing loss was noted. The main clinical features were presented in Table 3. The clinical diagnosis was molecularly confirmed, as we found a novel missense alteration c.83G>C p.(Arg28Pro) in the *TCOF1* gene (Figure 3A-C, Additional File 3).

**Family 3** **(sporadic case)**

Patient 3 was a sporadic male case born to a healthy, unrelated couple from the 2^nd^ uneventful pregnancy (1^st^ pregnancy was miscarried). Upon ultrasound examination, the index was prenatally diagnosed with a cleft palate. The birth parameters were as follows: body mass 3300 g (50^th^ percentile), length 52 cm (50^th^-75^th^ percentile), Apgar score was 10 at 1’. After birth, bilateral cleft of the soft palate, microphthalmia, micrognathia, bilateral microtia with an atresia of both external auditory canals were noted. The reconstruction of the palate was performed at the age of 8 months (Figure 3). The speech of the patient 3 was delayed, and it improved after bone conduction amplification at the age of 2.5 years. At the age of 7 years, the index presented with astigmatism with mild hyperopia and frequent infections of the upper airways. Furthermore, polysomnography revealed short and frequent obstructive apneas (16 per hour). CT scan of the ears showed hypoplastic ossicles and hypoplasia of the middle ear cavities. Upon clinical examination, micrognathia, hypoplasia of the lateral aspects of the orbits, down-slanting palpebral fissures, inverted lower palpebrae with sparse eyebrows, relatively high nasal bridge, dental misalignment, hypoplastic zygomatic bones, pectus excavatum and asymmetry of the scapulae were noted. The patient was clinically diagnosed with TCS, and the initial diagnosis was confirmed after revealing a novel variant c.4370delA p.(Lys1457Arg*fs**118) in the *TCOF1* gene (Additional File 3, Figure 3D-F).

**Family 4 (sporadic case)**

Patient 4 was a sporadic female born to a healthy, unrelated couple from the first twin pregnancy, in which one of the fetuses died antenatally at 9^th^ weeks of gestation. Upon ultrasound examination, the index was prenatally diagnosed with micro- and retrognathia. The patient was born naturally at 38^th^ weeks with the following parameters: body mass 2490 g (3^rd^ -10^th^ percentile), length 55 cm (above 97^th^ percentile), Apgar score 8-9-9 at 1-3-5’, respectively. After birth, abnormal face with extreme hypoplasia of the mandible, maxilla, and zygomatic arches was noted (Figure 4A,B). In addition, thumbs were hypoplastic, and there was limited elbow extension on both sides. Furthermore, bilateral stenosis of auditory canals was recognized. Abdominal ultrasound showed ectopic left kidney located in the pelvis, while the echocardiography was normal. The patient had delayed psychomotor development, with absent speech due to severe hypoplasia of the mandibulofacial structures. Clinical diagnosis in patient 4 was Nager syndrome. Conventional karyotyping showed normal female chromosomes: 46,XX. The patient died at the age of 22 months as a result of pneumonia after facial reconstructive surgery. The molecular diagnosis was done post-mortem and revealed a missense variant c.574G>T p.(Glu192*) in the *SF3B4* gene causing Nager syndrome (Figure 4C, Additional File 3)

**Family 5 (sporadic case)**

Patient 5 was a sporadic female case born from the second pregnancy to a healthy, unrelated couple. A healthy boy was delivered from the first pregnancy. The girl was born via a Cesarean section at 38^th^ weeks due to breech presentation and polyhydramnios with the following birth characteristics: body mass 3200 g (50^th^ percentile), length 51 cm (75^th^ percentile), HC 35 cm (75^th^ percentile), Apgar score 8-8-9 at 1-3-5’, respectively. The patient was born with tracheoesophageal atresia with lower fistula, which was operated on day 2. Clinical examination revealed bilateral syndactyly of toes 2-3-4-5 of the right foot and toes 2-3 of the left foot. CT imaging showed supernumerary bones in both feet (including additional metatarsal and phalangeal bones in the right foot and an irregular extra bone next to the II^nd^ metatarsal as well as absent phalanges in the left foot) (Figure 5 A-C). Echocardiography after birth showed patent foramen ovale (PFO), which spontaneously closed during the first year of life. An abdominal ultrasound scan was normal. In infancy, the patient presented with progressive bitemporal narrowing of the forehead and was diagnosed with a trigonocephaly at the age of 1.5 years. Afterwards, she underwent a neurosurgical procedure for the prematurely closed metopic suture. Brain MRI performed after surgical treatment showed a small venous hemangioma in the right frontal lobe. The hearing test, ophthalmological assessment, and EEG were all normal. Psychomotor and intellectual development, as well as body measurements at the age of 13 years, were entirely normal. Routine GTG-banding revealed a normal female karyotype: 46,XX. Because the patient was initially suspected of Feingold syndrome and Greig cephalopolysyndactyly, mutations within *MYCN* and *GLI3* were excluded. Surprisingly, targeted NGS revealed a novel missense mutation in the *EFTUD2* gene and the diagnosis was made based on the molecular result (Additional File 3, Figure 5D). The patient was affected by AFDGA.

**Family 6 (sporadic case)**

Patient 6 was a sporadic female case born to healthy, non-consanguineous parents from a third pregnancy. The first pregnancy ended up with a healthy daughter, while the second pregnancy ended in a spontaneous abortion. The patient was a girl born naturally at 40^th^ weeks with the following birth characteristics: body mass 3350 g (50-75^th^ percentile), length 60 cm (above 97^th^ percentile), HC 32 cm (3^rd^-10^th^ percentile), Apgar score 7-9-10 at 1-3-5’, respectively. Upon genetic evaluation at 6 weeks after birth, the following abnormalities were noted: muscular hypotonia, micrognathia, hypoplasia o the left zygomatic arches, hypoplastic left ear, preauricular pit and tag on the left side, down-slanting palpebral fissures, and dermoid cyst of the left eye (Figure 5 E-G). Echocardiography showed persistent ductus arteriosus (PDA) of 1.5 mm, which spontaneously closed during the first month. Since hearing tests performed at the age of 10 months showed moderate bilateral sensorineural hypoacusis, a hearing aid was introduced at the time of diagnosis. Psychomotor development was delayed, with independent walking achieved at the age of 2 years; speech was delayed as a consequence of congenital hearing impairment. At age 4.5, the girl was diagnosed with focal seizure disorder and microcephaly with HC of 45.5 cm (below 3^rd^ percentile). Abdominal ultrasound and brain MRI were normal. Upon recent clinical assessment at 6 years of age, the patient’s height and body mass were normal: 111.5 cm (10-25^th^ percentile) and 20.3 kg (50^th^ percentile). Conventional GTG-banding showed a normal female karyotype: 46,XX. Patient 6 was suspected of TCS however targeted NGS revealed a novel missense variant in the *EFTUD2* gene (Additional File 3, Figure 5H). The patient 1.6 was finally diagnosed with AFDGA

**Family 7 (sporadic case)**

Patient 7 was a 12 years old boy born from a third pregnancy to healthy and unrelated parents (1^st^ pregnancy was miscarried, while from the 2^nd^ one, a healthy girl was born). The boy was born naturally at 40^th^ weeks with the following birth parameters: body mass 2900 g (10^th^-25^th^ percentile), length 47 cm (25^th^ percentile), HC 33 cm (10^th^ percentile), Apgar score 10 at 1’. After birth, he was diagnosed with ventricular septal defect (VSD) of 7 mm in diameter and restriction of blood flow from both left and right ventricles. VSD was surgically corrected at the age of 3 years. In addition, the index had bilateral cleft lip and palate, bilateral inguinal hernia, and bilateral absence of thumbs as well as the absence of fifth digits of the hands and feet. Upon brain MRI, agenesis of the corpus callosum was detected. The ophthalmological assessment showed right-sided coloboma of the iris and retina. Psychomotor and intellectual development was normal. Genetic evaluation at the age of 12 years revealed pectus excavatum, limited extension of the elbow joints, triangular face, deep-set eyes, down-slanting palpebral fissures, relatively large bulbous nose, cup-shaped and low-set ears, maxillary hypoplasia and thin upper lip (Figure 6). The patient 7 was clinically diagnosed with Nager syndrome, but we found two compound heterozygous variants in the *DHODH* gene (Additional File 3, Figure 6). Hence, the final diagnosis was Miller syndrome that similarly to Nager syndrome belongs to AFDs (Table 1).

**Families 8-11**

All affected members from families no 8-11 (two sporadic and two familiar cases) were clinically diagnosed with Nager syndrome that belongs to AFD. Targeted NGS did not reveal any pathogenic variants that could cause the phenotype observed in diagnosed patients.
